# Polyphenol Compound as a Transcription Factor Inhibitor

**DOI:** 10.3390/nu7115445

**Published:** 2015-10-30

**Authors:** Seyeon Park

**Affiliations:** Department of Applied Chemistry, Dongduk Women’s University, Seoul 136-714, Korea; sypark21@dongduk.ac.kr; Tel.: +82-2940-4514

**Keywords:** transcription factor, c-jun/c-fos (AP-1), c-myc/max, NF-κB, β-catenin/Tcf, polyphenol

## Abstract

A target-based approach has been used to develop novel drugs in many therapeutic fields. In the final stage of intracellular signaling, transcription factor–DNA interactions are central to most biological processes and therefore represent a large and important class of targets for human therapeutics. Thus, we focused on the idea that the disruption of protein dimers and cognate DNA complexes could impair the transcriptional activation and cell transformation regulated by these proteins. Historically, natural products have been regarded as providing the primary leading compounds capable of modulating protein–protein or protein-DNA interactions. Although their mechanism of action is not fully defined, polyphenols including flavonoids were found to act mostly as site-directed small molecule inhibitors on signaling. There are many reports in the literature of screening initiatives suggesting improved drugs that can modulate the transcription factor interactions responsible for disease. In this review, we focus on polyphenol compound inhibitors against dimeric forms of transcription factor components of intracellular signaling pathways (for instance, c-jun/c-fos (Activator Protein-1; AP-1), c-myc/max, Nuclear factor kappa-light-chain-enhancer of activated B cells (NF-κB) and β-catenin/T cell factor (Tcf)).

## 1. Introduction

Polyphenol compounds have been widely studied for their bioactive properties as antioxidants and for their broad range of roles in the prevention of cancers, cardiovascular diseases and neurodegenerative diseases. Polyphenol compounds are abundant in numerous groups of natural plant products (fruit, vegetables, grains and nuts) and can be a dietary food constituent. Polyphenols are divided into several classes, *i.e.*, phenolic acids (hydroxybenzoic acids and hydroxycinnamic acids), flavonoids (flavonols, flavones, flavanols, flavanones, isoflavones, proanthocyanidins), stilbenes, and lignans [1,2,3].

The mechanism underlying how polyphenol compounds play roles in the prevention of cancers, cardiovascular diseases and neurodegenerative diseases may be related to their modulation of cell signaling pathways. Therefore, to explain the mechanisms behind the actions of polyphenols, a number of signaling targets have been studied.

In this review, we discuss in particular the disruption of multimeric forms of transcription factors such as c-jun/c-fos (Activator Protein-1; AP-1), c-myc/max, Nuclear factor kappa-light-chain-enhancer of activated B cells (NF-κB) and β-catenin/T cell factor (Tcf) with small polyphenol compounds that block their protein-protein interactions or their interactions with DNA.

Cell proliferation, differentiation and transformation are regulated in response to cell surface stimuli in accordance with intracellular signals. In the final stage of regulation, transcription factors play an important role in the expression of genetic information. AP-1 is a heterodimeric transcription factor formed by the products of the *fos* and *jun* proto-oncogene families. The fos and the jun proteins have almost identical amino acid sequences that comprise their basic DNA binding sequence (B) and the adjacent leucine zipper region (Zip), by which the proteins dimerize with each other [4,5,6]. The AP-1 transcription factor recognizes and binds specifically to the DNA sequence 5′-TGAG/CTCA, known as the AP-1 site [7,8]. In many cases, activation of c-jun is involved in transmitting cancer-promoting signals. The c-jun gene may be permanently activated or overexpressed, which can lead to neoplastic transformation [9,10]. Jun is known to be over-expressed between 4- and 12-fold in 40% of human small-cell lung cancers and 20% of non-small cell lung cancers [9]. In addition, jun may be involved in leukemia [11]. According to some reported research, the elevated levels of c-jun and c-fos expression, as well as of AP-1-dependent target genes, are found in tumors derived from *in vivo* and *in vitro* transformation [12,13]. Disruption of fos and jun dimerization has been shown to impair the transcriptional activation and cell transformation regulated by these proteins [14,15].

Similarly, the c-*myc* proto-oncogene product is one of the essential transcription factors that induce cellular growth, proliferation, cell cycle entry and differentiation and is believed to be involved in the generation of many types of human malignancies, cell cycle progression and proliferation [16,17,18,19]. Biological activity of myc occurs upon hetero-dimerization with max, a small and ubiquitously expressed phosphoprotein [20,21,22]. The C-terminal domain of the c-myc and max proteins includes a basic domain/helix-loop-helix/leucine zipper (b/HLH/Z) motif that mediates binding each other through the HLH/Z region and specific DNA recognition of CACGTG E box motifs present in all target genes through the basic domain [20,23,24]. Myc is constitutively overexpressed in lymphoblastoid cells lines derived from individuals with the cancer-prone condition Bloom’s syndrome and there is evidence that myc de-regulation may be involved in the early stages of mammary carcinogenesis [25,26,27]. Myc is enhanced in many tumors, particularly small-cell-lung, breast and cervical carcinomas [25,26,27,28]. Especially, amplified c-*myc* oncogene was found in human stomach cancers and it has been suggested that c-*myc* mRNA overexpression might be crucial in the early development of primary lesions as well as in the formation of metastatic lesions of carcinomas of the stomach [29,30].

Additionally, functional activation of β-catenin/T-cell factor (Tcf) signaling has been implicated in human carcinogenesis. In cytoplasm, β-catenin contributes to cell-cell adhesion in cooperation with the cytoplasmic domain of E-cadherin, but β-catenin moves into the nucleus and possesses transcriptional activity in cooperation with the T-cell factor (Tcf)/lymphoid enhancer factor (Lef) transcription factor [31]. Activated β-catenin/Tcf signaling by the accumulation of β-catenin in the nucleus has been implicated in human carcinogenesis including colorectal cancer (CRC), melanoma, hepatocellular carcinoma, and gastric carcinoma [32,33,34]. One adenomatous polyposis coli (APC) mutation is observed in at least 60% of sporadic CRC cases and abnormalities in both APC alleles are shown in almost 30% of such cases [35]. Studies have reported the detection of APC mutations in 12 of 46 gastric cancers, with β-catenin nuclear localization occurring in both diffuse- and intestinal-type gastric cancers at a higher rate [36,37]. This means that the dysregulation of β-catenin plays a crucial role in some cancer cells. Thus, oncogenic transcription factors such as AP-1, myc-max and β-catenin/Tcf may present promising targets for cancer prevention.

NF-κB is also a protein complex transcription factor comprised of p50 and p65 or Rel. NF-κB is involved in cellular responses to stimuli such as oxidative stress and cytokines [38]. NF-kB is constitutively active in several cancer types and has been associated with the regulation of cell proliferation, cell survival, invasion, metastasis and inhibition of apoptosis [39,40]. It has been suggested that inhibition of NF-kB signaling suppresses tumor formation [41,42,43].

## 2. Symmetric Polyphenols

Curcumin, with a distinct symmetric polyphenol structure, is known to have diverse biological activities including antiinflammatory, antitumor, antioxidant, antifungal and antibacterial actions. Recently, a daily dose of curcumin was recommended for cancer patients [44]. In addition to curcumin, other types of symmetric polyphenols like dihydroguaiaretic acid (DHGA) and nordihydroguaiaretic acid (NDGA) blocked transcription factor and DNA binding (Table 1). Each bZIP domain of the jun and fos proteins used in electrophoresis mobility shift assay (EMSA) contain only a basic region and a leucine zipper region, not including dephosphorylation and phosphorylation sites activated by protein kinase C (PKC) and Jun N-terminal kinase (JNK) or Fyn-related kinase (FRK), respectively [45,46]. Thus, the possible inhibition sites of these inhibitors are limited to both basic regions and the leucine zippers of c-jun and c-fos proteins. To investigate whether functional group modification of the benzaldehyde moiety in the curcuminoid structure affects the inhibition of fos-jun and DNA complex formation, our group synthesized a variety of curcuminoids as shown in Table 2. Curcumin and some synthetic curcumin derivatives were found to be transcription factor inhibitors following EMSA experiments using *in vitro* expressed c-fos and c-jun proteins. The inhibitory actions of curcumin, curcuminoids, DHGA and NDGA on jun/AP-1 and DNA binding in cell nuclear extracts may be not via blocking the regulatory domain at c-jun sites that negatively regulate its DNA binding activity, but via direct interference with dimer binding to DNA. The inhibitors showed an inhibitory effect against the formation of the fos–jun–DNA complex in an *in vitro* assay with the IC_50_ values shown in Table 1 and Table 2. Curcumin and NDGA were revealed to have an IC_50_ value of around 0.3 mM in an *in vitro* assay, showing a more potent inhibitory effect on the formation of the fos–jun–DNA complex than DHGA. The change of the methoxy functional group of DHGA into a hydroxy group of NDGA caused a marked increase in the inhibitory activity. Remarkably, the synthetic curcumin derivative BJC005 showed the highest inhibitory activity out of all the synthetic curcuminoids tested. BJC005, with an IC_50_ of 5.4 μM, shows a much greater inhibitory effect than momordin, reported hitherto as the best fos-jun-DNA complex inhibitor [44,47]. However, some curcuminoids, such as CHC006 and BJC004, showed no inhibitory activity against fos-jun-DNA complex formation [44].

The myc and max proteins, containing basic regions and helix-loop-helix-zipper regions, were over-expressed in *E*. *coli* and used for the investigation into the inhibitors of myc-max-DNA complex formation. Curcuminoid 004, 005, and 008 demonstrated relatively selective inhibition of myc-max-DNA binding *versus* jun-fos-DNA complex formation. When assayed using the myc-max dimer protein expressed in *E. coli*, NDGA showed a weak inhibitory effect on the dimer and cognate DNA complex formation (~8 mM of IC_50_). This value represents about 50-fold specificity of NDGA to AP-1. However, the inhibitory effect of NDGA is more specific to β-catenin/Tcf than AP-1 or myc-max transcription factor [48,49]. This suggests that polyphenol compounds can be selective inhibitors against each transcription activator, therefore may be effective modulators of cell signaling and regulation.

Altogether, these results suggest that the substituted position of the benzaldehyde moiety and the polarity property of the substituted group play an important role in the inhibition. Regarding the inhibitory activity of curcuminoids on AP-1, p-position- and polar group-substituted curcuminoids have a tendency to inhibit the fos-jun-DNA complex formation more potently [44]. Considering that the insertion of a nitro group into the benzene ring at CHC 007, 008, 009, 011 and BJC005 contribute to efficient inhibitory activity, the substitution of polar groups in benzene rings is likely to confer a richly negative charge to the molecule itself, making them potent competitors to DNA for interaction with the protein.

**Figure 1:**
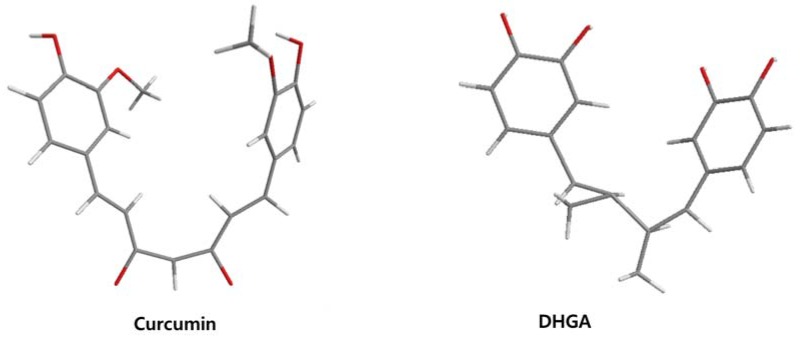
The lowest energy conformation of curcumin and DHGA.

The low energy conformations of curcumin and DHGA inhibitors have “V-shaped” two-fold symmetry as shown in Figure 1 [49]. Conformational studies of these inhibitors revealed that they share a common conformational feature. The two-fold symmetry of molecular conformation is also a characteristic of fos-jun or myc-max transcription activators that function as dimer proteins. As a result, a stable binding mode was proposed due to two potential interactions between the protein and the inhibitor. One is a hydrogen bond and/or an electrostatic interaction between the acidic hydroxyl (OH)-group in the phenol ring of the inhibitor and the amino-group of the basic amino acid, which is abundant in the DNA binding region of the dimeric forms of the transcription factor. The other is a hydrophobic interaction between the hydrocarbon chain of the inhibitor and the hydrocarbon chain of the amino acid in the fos-jun protein [49]. The inhibitors have differences in their molecular structures. DHGA and NDGA have the same hydrophobic carbon chain. DHGA has one phenolic OH and one methoxy group on each side while NDGA has two phenolic OH groups on each side. The methoxy group of DHGA cannot form hydrogen bonds. This might make DHGA a less potent inhibitor than NDGA. Although curcumin has one phenolic OH on each side, the conjugated double bond of the hydrophobic carbon chain may cause curcumin to be more hydrophobic than the other two inhibitors. It was also suggested that the rigid conformation of curcumin provides a better acidic OH and hydrocarbon orientation for interactions with the fos-jun protein [49]. Actually, the lowest interaction energy for each inhibitor, which was obtained using Monte Carlo docking simulations, showed a correlation with the IC_50_ value of each inhibitor, which was experimentally determined.

**Table 1:** Structures and activities of polyphenol inhibitors against c-jun/c-fos (AP-1).

Name	Structure	IC_50_ (mM)		
		AP-1	Myc/Max	β-catenin
**DHGA**	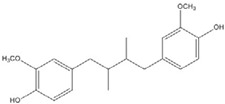	8.4 [45]		
**NDGA**	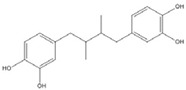	0.34 [45]	8 [49]	0.01 [48]
**Curcumin**	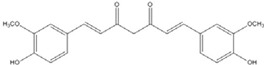	0.28 [45]	0.6 [49]	0.012 [46]
**Asarinin**	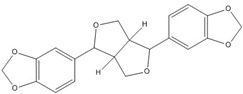		0.3 [49]	
**Resveratrol**	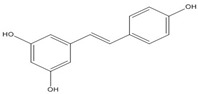	0.03 [50,51]		
**T-5224**	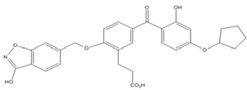	2 [52]		
**Anthraquinone**	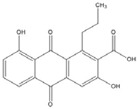	0.1 [53]		

DHGA: dihydroguaiaretic acid; NDGA: nordihydroguaiaretic acid; AP-1: Activator Protein-1.

Resveratrol and anthraquinone derivatives were also shown to inhibit the binding of AP-1 to the AP-1 binding site [50,51]. A new benzophenone derivative T-5224 was found to inhibit the DNA-binding activity of c-fos/c-jun, with IC_50_ values around 2 mM [52,53]. Although IC_50_ values around 2 mM is not very efficient, this compound exhibited a good specificity profile since the small-molecule did not affect the activities of other transcription factors including CCAAT-enhancer-binding proteins (C/EBP) and Activating Transcription Factor 2 (ATF-2) (bZIP domain) as well as MyoD (bHLH domain).

**Table 2:** Structures and activities of curcuminoid inhibitors against transcription factors.

Name	Structure		IC_50_ (mM)	
		Activator Protein-1	Myc/Max	β-Catenin
**Curcumin**	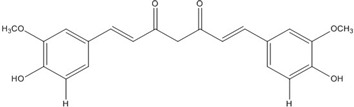	0.28 [44]	0.6 [49]	0.012 [54]
**CHC001**	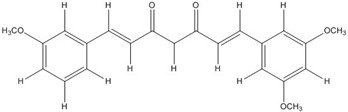	5.4 [44]		
**CHC002**	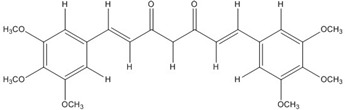	6.7 [44]		
**CHC003**	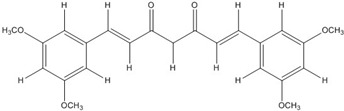	3.9 [44]		
**CHC004**	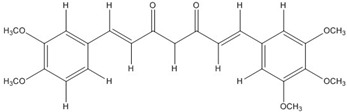	1.6 [44]	0.082 [49]	
**CHC005**	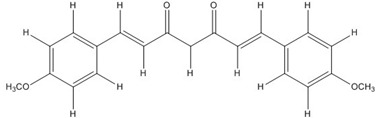	1.8 [44]	0.4 [49]	
**CHC006**	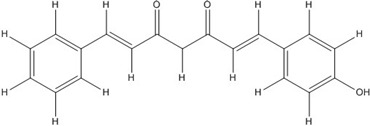	No inhibition [44]		
**CHC007**	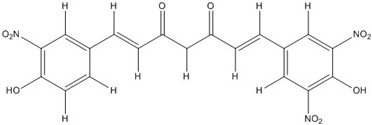	0.38 [44]		0.06 [54]
**CHC008**	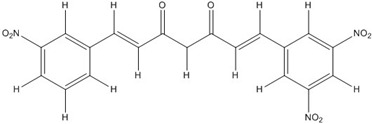	1.4 [44]	0.16 [49]	
**CHC009**	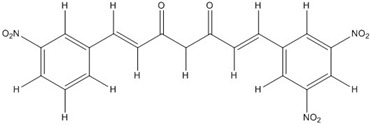	0.64 [44]		
**CHC011**	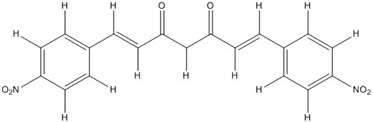	0.3 [44]	0.43 [49]	
**BJC004**	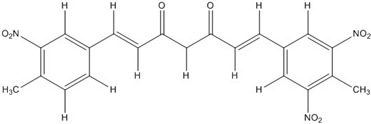	No inhibition [44]		
**BJC005**	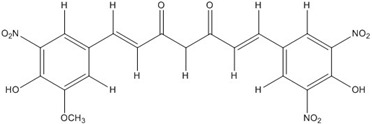	0.0054 [44]		

Curcumin and synthetic curcumin derivatives were also suggested to be β-catenin/Tcf signaling inhibitors [54,55,56]. In our previous reports, curcumin and some synthetic derivatives inhibited β-catenin/Tcf signaling in cell-based luciferase assays and their inhibitory mechanisms were found to be related to the reduced binding of nuclear β-catenin and Tcf-4 proteins [54]. 20 μM curcumin and 100 μM CHC007 inhibited β-catenin/Tcf signaling by 58%–63% and 70%–78%, respectively, compared with the control showing variations in a variety of cell lines [54].

## 3. Flavonoids

Many flavonoid target pathways related to intracellular signaling have been suggested and reviewed [57]. Although flavonoids were suggested as potential wnt/beta-catenin signaling modulators in cancer, all of the flavonoids did not show the same mechanism. Also, some compounds showed different mechanisms in different component targets. For instance, flavonoids like genistein, tangeretin, epigallocathechin gallate (EGCG), quercetin, apigenin and isoquercitrin were reported to modulate wnt/β-catenin signaling [57,58,59,60,61,62,63,64]. But these flavonoids do not all affect the same targets. Genistein seems to inhibit not only wnt/receptor binding but also Akt action, leading to β-catenin activation [54,65]. Moreover, genistein can inhibit the binding of β-catenin/Tcf complexes with consensus DNA [66]. Quercetin not only targets Akt and MAPK but also interferes with the binding of Tcf complexes to DNA [57,65,67,68]. Flavonoid compounds that disrupt the transcription factor complex are listed in Table 3.

Common flavonols including quercetin, kaempferol, and isorhamnetin as well as flavones such as baicalein and one of several known isoflavones, genistein, showed inhibitory effects in β-catenin activated cells [66,67]. The mechanism underlying the reduction of β-catenin/Tcf transcriptional activity by quercetin, kaempferol, isorhamnentin, baicalein and genistein was due to the decreased binding of β-catenin/Tcf complexes with consensus DNA. EMSA data showed that the binding of Tcf complexes to DNA was impaired by inhibitors. Conforming to these results, coimmunoprecipitation of β-catenin and Tcf-4 using Tcf-4 antibodies showed that the association of β-catenin with Tcf-4 was blocked by inhibitors. With genistein, the decreased distribution of β-catenin in the nucleus caused by decreased phosphorylation of Akt and GSK3b contributed as a partial mechanism for reduced β-catenin/Tcf transcriptional activity [66]. Thus, these results agree with the earlier reports by Jaiswal *et al.* and Sarkar *et al.* [54,65].

**Table 3:** Structures and activities of flavonoid inhibitors against transcription factors.

Name	Structure	AP-1	IC50 (mM) Myc/Max	β-catenin	NF-κB
**Quercetin**	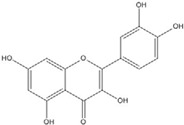			0.0076 [67]	0.03 [69]
**Genistein**	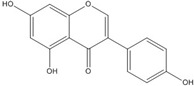			0.01 [66]	
**Kaempferol**	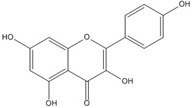	0.0029 [49]	2 [49]	0.0016 [66]	0.003 [70]
**Isorhamnetin**	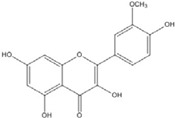			0.0062 [66]	
**Baicalein**	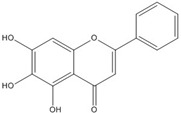			0.00026 [66]	
**Naringenin**	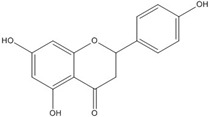			No inhibition [71]	
**Epigallo catechingallate**	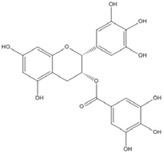			No inhibition [49]	
**Sterptonigrin**	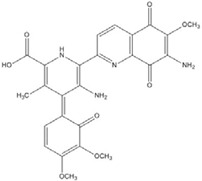			0.005 [72]	
**Tanshinone I**	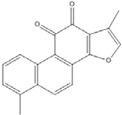	2.6 [49]			
**7,8-dihydroxy flavanone**	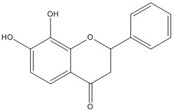	1.8 [49]			
**Chrysin**	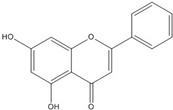	0.2 [53]			
**Apigenin**	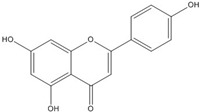	0.1 [53]			~0.002 [53]
**Luteolin**	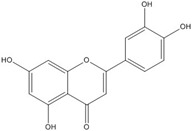	0.1 [53]			~0.002 [53]
**Flavanone**	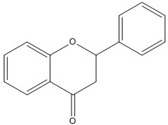			No inhibition [71]	>0.003 [53]

AP-1: Activator Protein-1; NF-κB : Nuclear factor kappa-light-chain-enhancer of activated B cells.

Although flavonoids such as 7,8-dihydroxyflavanone showed inhibitory activity against jun-fos/DNA binding, the inhibitory action was not as effective as on β-catenin/Tcf, with a 1.8 mM IC_50_ value obtained from EMSA [49]. Although there are few reports on the inhibitory effect of flavonoids against AP-1 or other transcription factors, our unpublished data showed that kaempferol inhibited fos-jun-DNA binding and myc-max-DNA binding with IC_50_ values of 2.9 mM and 2 mM, respectively [49]. Also, tanshinone I and 7,8 dihydroxyflavanone showed inhibitory activities against fos-jun-DNA binding, with IC_50_ values of 2.6 and 1.8 mM, respectively [49]. All these data demonstrate that flavonoids show selective inhibitory activity against β-catenin/Tcf rather than against the AP-1 transcription factor. However, quercetin, kaempferol, apigenin, luteolin and chrysin were reported to inhibit the NF-kB transcription factor by interfering with complex formation [69,70]. Interestingly, flavanone and flavonoids that include a flavanone skeleton such as naringenin and EGCG showed no inhibitory activity on the direct binding of β-catenin/Tcf with DNA ([49,68,71].

Although genistein, kaempferol, isorhamnentin and baicalein possess a common phenylbenzopyrone structure (C6–C3–C6), they are mainly categorized into flavanols (kaempferol and isorhamnentin), flavone (baicalein), and isoflavone (genistein) according to the substitution state and direction of the phenyl ring. Genistein showed the least potent blocking activity in EMSA while baicalein showed the most [66]. Considering the relationship between the structure and β-catenin/Tcf inhibitory activity, the direction of the B phenyl ring at the 2-subtituted site (flavanol and flavone) appears to be more effective than at the 3-substituted (isoflavone) site. It also seems that hydroxyl group substitution at the 3-site (flavanol) reduces the potency of inhibition compared to without substitution (flavone).

**Figure 2:**
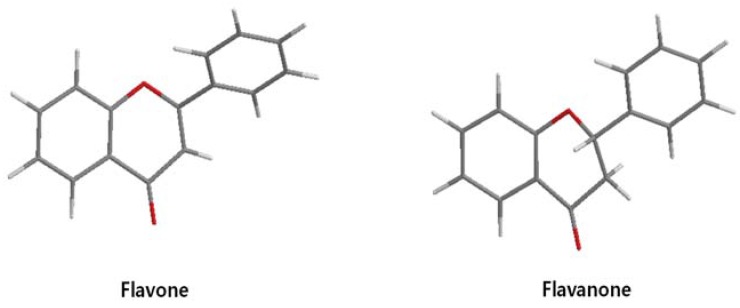
Flavone and flavanone.

On the other hand, flavanone and flavonoids that include the same skeleton such as naringenin did not show inhibitory activity against β-catenin/Tcf and DNA binding, in discord with other types of flavonoids [68,71]. From the three dimensional structure, the 2-substituted B ring of flavanone seems to be puckered from the plane, with a different shape from the other flavone skeletons containing conjugated double bonds of C3 rings as shown in Figure 2. This puckered structure may be unfit to block β-catenin/Tcf and DNA complex formation.

## 4. Non Flavonoid Polyphenol β-catenin Inhibitors

Because wnt/β-catenin signaling is involved in a broad range of biological systems, small molecular inhibitors of wnt/β-catenin signaling have received attention as targets for therapeutic interventions. A variety of inhibitors against other components in wnt/β-catenin signaling were recently reviewed [73]. Out of several small molecules, polyphenol compounds that disrupt the β-catenin/Tcf-4 complex are listed in Table 4.

**Table 4:** Structures and activities of polyphenol inhibitors against β-catenin/Tcf.

Compound	Structure	β-Catenin IC_50_ (μM)
PKF118-744	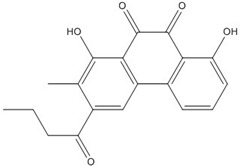	2.4 [73,74,75,76]
CGP049090	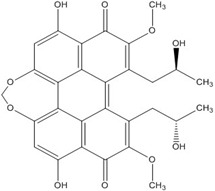	8.7 [73,74,75,76]
PKF118-310	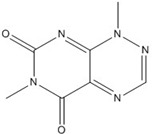	0.8 [73,74,75,76]
ZTM00990	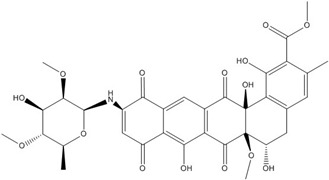	0.64 [73,74,75,76]
BC21	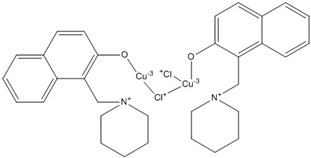	Not Determined [73,77]
Ethacrynic acid	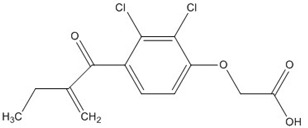	~70 [73,78,79]
Ethacrynic acid derivative	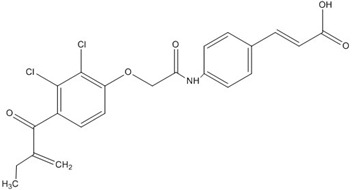	~5 [73,79]
PKF115-584	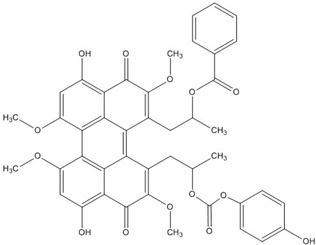	3.2 [73,74,75,76]
PNU-74654	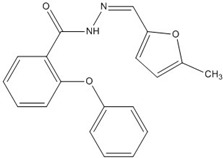	Not Determined [73,74,75,76]

## 5. Conclusions

A useful starting point for investigations into target-specific drug design is to identify effective targets that elicit responses in physiological states and to compile an abundant candidate library of effective leading compounds. Polyphenol compounds are abundant and ideally include more comprehensive structural variance, with effects on intracellular signaling induced by the altered responsiveness (for instance, inhibition against tumor necrosis factors (TNF)-induced, transforming growth factor (TGF)-induced, α-melanocyte-stimulating hormone (α-MSH)-induced, oxidative stress-induced and UV-induced signaling).

A general concern is that many of the targets for intracellular signaling may play vital roles in other signaling cascades. Moreover, a critical issue that should be addressed is whether targeting of the dimeric forms of transcription factors is sufficiently specific. For instance, AP-1, myc-max, NF-κB and β-catenin/Tcf transcription factors play specific roles in a variety of signaling pathways. Therefore, broad inhibition of the transcription factors above is predicted to cause toxicity; this is why specificity will be an important determinant of the success of transcription factor inhibiting drugs. Thus, whether the inhibition of AP-1, myc-max, NF-κB and β-catenin/Tcf can be discriminated by chemical inhibitors remains to be explored. Theoretically, DNA can form very large interfaces with proteins and the buried surfaces of the protein–nucleic acid interfaces have a broad range of sizes [80]. Usually, protein–protein recognition sites are formed by protein surfaces with good shape and electrostatic complementarity [81]. Protein–protein interfaces are frequently hydrophobic and bury a large portion of the nonpolar surface area [82]. However, in protein-DNA interaction, the DNA binding surface of the protein harbors basic amino acids because of the negative charge of DNA. It has been reported that standard sizes for the interface range from 1200–2000 A˚ [83]. The interface between transcription factor and DNA is enough for multi-facial binding of polyphenol compounds showing approximate sizes from 7 to 10 A˚.

This review discusses polyphenol compounds with roles in direct inhibition against transcription factor complexes such as AP-1, myc-max, NF-κB and β-catenin/Tcf and suggests structure-activity relationships for the development of small-molecule drug candidates that modulate this clinically relevant signaling pathway. The structural diversity of curcumin derivative compounds or flavonoids may provide specificity for recognizing transcription factors. Polyphenol groups have structural variability and abundance in nature and show distinctive inhibitory activities against various transcription factors. Altogether, polyphenol transcription factor inhibitors have significant value as clinical drug candidates. However, another aspect that has to be considered is that some polyphenol compounds that reach cells and tissues are chemically different from the original dietary form because of structural modifications due to the conjugation process that takes place in the small intestine and, mostly, in the liver [84]. As animal trials are performed in future studies, they will provide extremely valuable information and overcome some of the difficulties associated with bioavailability in humans as clinical drug candidates.

## References

[1] Manach C., Scalbert A., Morand C., Rémésy C., Jiménez L. (2004). Polyphenols: Food sources and bioavailability. Am. J. Clin. Nutr..

[2] Manach C., Williamson G., Morand C., Scalbert A., Rémésy C. (2005). Bioavailability and bioefficacy of polyphenols in humans. I. Review of 97 bioavailability studies. Am. J. Clin. Nutr..

[3] El Gharras H. (2009). Polyphenols: Food sources, properties and applications—A review. Int. J. Food Sci. Tech..

[4] Curran T., Franza B.R. (1988). Fos and Jun: The AP-1 connection. Cell.

[5] Johnson P.F., McKnight S.L. (1989). Eukaryotic transcriptional regulatory proteins. Annu. Rev. Biochem..

[6] Mitchell P.J., Tjian R. (1989). Transcriptional regulation in mammalian cells by sequence-specific DNA binding proteins. Science.

[7] Lee W., Mitchell P., Tjian R. (1987). Purified transcription factor AP-1 interacts with TPA-inducible enhancer elements. Cell.

[8] Angel P., Imagawa M., Chiu R., Stein B., Imbra R.J., Rahmsdorf H.J., Jonat C., Herrlich P., Karin M. (1987). Phorbol ester-inducible genes contain a common cis element recognized by a TPA-modulated trans-acting factor. Cell.

[9] Schutte J., Nau M., Birrer M., Thomas F., Gazdar A., Minna J. (1988). Constitutive expression of multiple mRNA forms of the c-jun oncogene in human lung cancer cell lines. Proc. Am. Assoc. Cancer Res..

[10] Bossy-Wetzel E., Bravo R., Hanahan D. (1992). Transcription factors junB and c-jun are selectively up-regulated and functionally implicated in fibrosarcoma development. Genes Dev..

[11] Mattei M.G., Simon-Chazottes D., Hirai S., Ryseck R.P., Galcheva-Gargova Z., Guénet J.L., Mattei J.F., Bravo R., Yaniv M. (1990). Chromosomal localization of the three members of the jun proto-oncogene family in mouse and man. Oncogene.

[12] Schutte J., Minna J.D., Birrer M.J. (1989). Deregulated expression of human c-jun transforms primary rat embryo cells in cooperation with an activated c-Ha-ras gene and transforms Rat-1a cells as a single gene. Proc. Natl. Acad. Sci. USA.

[13] Van der Burg B., van Selm-Miltenburg A.J.P., de Laat S.W., van Zoolen F.J.J. (1989). Direct effects of estrogen on c-fos and c-myc protooncogene expression and cellular proliferation in human mammary breast cancer cells. Mol. Cell. Endocrinol..

[14] Baichwal V.R., Tjian R. (1990). Control of c-Jun activity by interaction of a cell-specific inhibitor with regulatory domain delta: Differences between v- and c-Jun. Cell.

[15] Persson A.E., Pontén I., Cotgreave I., Jernström B. (1996). Inhibitory effects on the DNA binding of AP-1 transcription factor to an AP-1 binding site modified by benzo[α]pyrene 7,8-dihydrodiol 9,10-epoxide diastereomers. Carcinogenesis.

[16] Dalla-Favera R., Wong-Staal F., Gallo R.C. (1982). Onc gene amplification in promyelocytic leukaemia cell line HL-60 and primary leukaemic cells of the same patient. Nature.

[17] Magrath I. (1990). The pathogenesis of Burkitt’s lymphoma. Adv. Cancer Res..

[18] Payne G.S., Bishop J.M., Varmus H.E. (1982). Multiple arrangements of viral DNA and an activated host oncogene in bursal lymphomas. Nature.

[19] Neil J.C., Forrest D., Doggett D.L., Mullins J.I. (1987). The role of feline leukaemia virus in naturally occurring leukaemias. Cancer Surviv..

[20] Eilers M., Schirm S., Bishop J.M. (1991). The MYC protein activates transcription of the alpha-prothymosin gene. EMBO J..

[21] Penn L.J., Laufer E.M., Land H. (1990). C-MYC: Evidence for multiple regulatory functions. Semin. Cancer Biol..

[22] Land H., Parada L.F., Weinberg R.A. (1983). Tumorigenic conversion of primary embryo fibroblasts requires at least two cooperating oncogenes. Nature.

[23] Coppola J.A., Cole M.D. (1986). Constitutive c-myc oncogene expression blocks mouse erythroleukaemia cell differentiation but not commitment. Nature.

[24] Miner J.H., Wold B.J. (1991). c-myc inhibition of MyoD and myogenin-initiated myogenic differentiation. Mol. Cell. Biol..

[25] Little C.D., Nau M.M., Carney D.N., Gazdar A.F., Minna J.D. (1983). Amplification and expression of the c-myc oncogene in human lung cancer cell lines. Nature.

[26] Escot C., Theillet C., Lidereau R., Spyratos F., Champeme M.H., Gest J., Callahan R. (1986). Genetic alteration of the c-myc protooncogene (MYC) in human primary breast carcinomas. Proc. Natl. Acad. Sci. USA.

[27] Pinion S.B., Kennedy J.H., Miller R.W., MacLean A.B. (1991). Oncogene expression in cervical intraepithelial neoplasia and invasive cancer of cervix. Lancet.

[28] Kim Y.S., Yoo H.S., Lee K.T., Goh S.H., Jung J.S., Oh S.W., Baba M., Yasuda T., Matsubara K., Nagai H. (2000). Detection of genetic alterations in the human gastric cancer cell lines by two-dimensional analysis of genomic DNA. Int. J. Oncol..

[29] Morgenbesser S.D., DePinho R.A. (1994). Use of transgenic mice to study myc family gene function in normal mammalian development and in cancer. Semin. Cancer Biol..

[30] Pelengaris S., Rudolph B., Littlewood T. (2000). Action of Myc *in vivo*—Proliferation and apoptosis. Curr. Opin. Genet. Dev..

[31] Bullions L.C., Levine A.J. (1998). The role of beta-catenin in cell adhesion, signal transduction, and cancer. Curr. Opin. Oncol..

[32] Morin P.J., Sparks A.B., Korinek V., Barker N., Clevers H., Vogelstein B., Kinzler K.W. (1997). Activation of b-catenin-Tcf signaling in colon cancer by mutations in β-catenin or APC. Science.

[33] Fujie H., Moriya K., Shintani Y., Tsutsumi T., Takayama T., Makuuchi M., Kimura S., Koike K. (2001). Frequent β-catenin aberration in human hepatocellular carcinoma. Hepatol. Res..

[34] Woo D.K., Kim H.S., Lee H.S., Kang Y.H., Yang H.K., Kim W.H. (2001). Altered expression and mutation of β-catenin gene in gastric carcinomas and cell lines. Int. J. Cancer.

[35] Powell S.M., Zilz N., Beazer-Barclay Y., Bryan T.M., Hamilton S.R., Thibodeau S.N., Vogelstein B., Kinzler K.W. (1992). APC mutations occur early during colorectal tumorigenesis. Nature.

[36] Nakatsuru S., Yanagisawa A., Ichii S., Tahara E., Kato Y., Nakamura Y., Horii A. (1992). Somatic mutation of the APC gene in gastric cancer: Frequent mutations in very well differentiated adenocarcinoma and signet-ring cell carcinoma. Hum. Mol. Genet..

[37] Clements W.M., Wang J., Sarnaik A., Kim O.J., MacDonald J., Fenoglio-Preiser C., Groden J., Lowy A.M. (2002). β-catenin mutation is a frequent cause of Wnt pathway activation in gastric cancer. Cancer Res..

[38] Tian B., Brasier A.R. (2003). Identification of a nuclear factor κB-dependent gene network. Recent Prog. Horm. Res..

[39] Karin M., Cao Y., Greten F.R., Li Z.W. (2002). NF-κB in cancer: From innocent bystander to major culprit. Nat. Rev. Cancer.

[40] Mees C., Nemunaitis J., Senzer N. (2009). Transcription factors: Their potential as targets for an individualized therapeutic approach to cancer. Cancer Gene Ther..

[41] Xiong H.Q., Abbruzzese J.L., Lin E., Wang L., Zheng L., Xie K. (2004). NF-κB activity blockade impairs the angiogenic potential of human pancreatic cancer cells. Int. J. Cancer.

[42] Brickman J.M., Adam M., Ptashne M. (1999). Interactions between an HMG-1 protein and members of the Rel family. Proc. Natl. Acad. Sci. USA.

[43] Campisi J. (2001). Cellular senescence as a tumor suppressor mechanism. Trends Cell Biol..

[44] Hahm E.R., Cheon G., Lee J., Kim B., Park C., Yang C.H. (2002). New and known symmetrical curcumin derivatives inhibit the formation of Fos-Jun-DNA complex. Cancer Lett..

[45] Park S., Lee D.K., Yang C.H. (1998). Inhibition of fos-jun-DNA complex formation by dihydroguaiaretic acid and *in vitro* cytotoxic effects on cancer cells. Cancer Lett..

[46] Hahm E.R., Gho Y.S., Park S., Park C., Kim K.W., Yang C.H. (2004). Synthetic curcumin analogs inhibit activator protein-1 transcription and tumor-induced angiogenesis. Biochem. Biophys. Res. Commun..

[47] Lee D.K., Kim B., Lee S.G., Gwon H.J., Moon E.Y., Hwang H.S., Seong S.K., Lee M., Lim M.J., Sung H.J. (1998). Momordins inhibit both AP-1 function and cell proliferation. Anticancer Res..

[48] Park S., Lee J. (2011). Inhibitory effect of nordihydroguaiaretic acid on β-catenin/Tcf signalling in β-catenin-activated cells. Cell Biochem. Funct..

[49] Park S. (2002). Studies on the Inhibitory Mechanism of the Dimeric Forms of Transcription Activators. Ph.D. Thesis.

[50] Yu R., Hebbar V., Kim D.W., Mandlekar S., Pezzuto J.M., Kong A.N. (2001). Resveratrol inhibits phorbol ester and UV-induced activator protein 1 activation by interfering with mitogen-activated protein kinase pathways. Mol. Pharmacol..

[51] Goto M., Masegi M., Yamauchi T., Chiba K., Kuboi Y., Harada K., Naruse N. (1998). K1115 A, a new anthraquinone derivative that inhibits the binding of activator protein-1 (AP-1) to its recognition sites. I. Biological. activities. J. Antibiot..

[52] Aikawa Y., Morimoto K., Yamamoto T., Chaki H., Hashiramoto A., Narita H., Hirono S., Shiozawa S. (2008). Treatment of arthritis with a selective inhibitor of c-Fos/activator protein-1. Nat. Biotechnol..

[53] Yap J.L., Chauhan J., Jung K.Y., Chen L., Prochownik E.V., Fletcher S. (2012). Small-molecule inhibitors of dimeric transcription factors: Antagonism of protein–protein and protein–DNA interactions. Med. Chem. Commun..

[54] Park C.H., Hahm E.R., Park S., Kim H.K., Yang C.H. (2005). The inhibitory mechanism of curcumin and its derivative against beta-catenin/Tcf signaling. FEBS Lett..

[55] Jaiswal A.S., Marlow B.P., Gupta N., Narayan S. (2002). Beta-catenin-mediated transactivation and cell-cell adhesion pathways are important in curcumin (diferuylmethane)-induced growth arrest and apoptosis in colon cancer cells. Oncogene.

[56] Mahmoud N.N., Carothers A.M., Grunberger D., Bilinski R.T., Churchill M.R., Martucci C., Newmark H.L., Bertagnolli M.M. (2000). Plant phenolics decrease intestinal tumors in an animal model of familial adenomatous polyposis. Carcinogenesis.

[57] Amado N.G., Fonseca B.F., Cerqueira D.M., Neto V.M., Abreu J.G. (2011). Flavonoids: Potential Wnt/beta-catenin signaling modulators in cancer. Life Sci..

[58] Song D.H., Sussman D.J., Seldin D.C. (2000). Endogenous protein kinase CK2 participates in Wnt signaling in mammary epithelial cells. J. Biol. Chem..

[59] Landesman-Bollag E., Song D.H., Romieu-Mourez R., Sussman D.J., Cardiff R.D., Sonenshein G.E., Seldin D.C. (2001). Protein kinase CK2: Signaling and tumorigenesis in the mammary gland. Mol. Cell. Biochem..

[60] Kim J., Zhang X., Rieger-Christ K.M., Summerhayes I.C., Wazer D.E., Paulson K.E., Yee A.S. (2006). Suppression of Wnt signaling by the green tea compound (−)−epigallocatechin 3-gallate (EGCG) in invasive breast cancer cells. Requirement of the transcriptional repressor HBP1. J. Biol. Chem..

[61] Dashwood W.M., Orner G.A., Dashwood R.H. (2002). Inhibition of beta-catenin/Tcf activity by white tea, green tea, and epigallocatechin-3-gallate (EGCG): Minor contribution of H_2_O_2_ at physiologically relevant EGCG concentrations. Biochem. Biophys. Res. Commun..

[62] Pahlke G., Ngiewih Y., Kern M., Jakobs S., Marko D., Eisenbrand G. (2006). Impact of quercetin and EGCG on key elements of the Wnt pathway in human colon carcinoma cells. J. Agric. Food Chem..

[63] Gao Z., Xu Z., Hung M.S., Lin Y.C., Wang T., Gong M., Zhi X., Jablon D.M., You L. (2009). Promoter demethylation of WIF-1 by epigallocatechin-3-gallate in lung cancer cells. Anticancer Res..

[64] Mount J.G., Muzylak M., Allen S., Althnaian T., McGonnell I.M., Price J.S. (2006). Evidence that the canonical Wnt signalling pathway regulates deer antler regeneration. Dev. Dyn..

[65] Sarkar F.H., Li Y., Wang Z., Kong D. (2009). Cellular signaling perturbation by natural products. Cell Signal..

[66] Park S., Choi J. (2010). Inhibition of beta-catenin/Tcf signaling by flavonoids. J. Cell. Biochem..

[67] Park C.H., Chang J.Y., Hahm E.R., Park S., Kim H.K., Yang C.H. (2005). Quercetin, a potent inhibitor against beta-catenin/Tcf signaling in SW480 colon cancer cells. Biochem. Biophys. Res. Commun..

[68] Park C.H., Hahm E.R., Lee J.H., Jung K.C., Yang C.H. (2005). Inhibition of beta-catenin-mediated transactivation by flavanone in AGS gastric cancer cells. Biochem. Biophys. Res. Commun..

[69] Min Y.D., Choi C.H., Bark H., Son H.Y., Park H.H., Lee S., Park J.W., Park E.K., Shin H.I., Kim S.H. (2007). Quercetin inhibits expression of inflammatory cytokines through attenuation of NF-κB and p38 MAPK in HMC-1 human mast cell line. Inflamm. Res..

[70] Chen C.C., Chow M.P., Huang W.C., Lin Y.C., Chang Y.J. (2004). Flavonoids inhibit tumor necrosis factor-alpha-induced up-regulation of intercellular adhesion molecule-1 (ICAM-1) in respiratory epithelial cells through activator protein-1 and nuclear factor-κB: Structure-activity relationships. Mol. Pharmacol..

[71] Lee J.H., Park C.H., Jung K.C., Rhee H.S., Yang C.H. (2005). Negative regulation of β-catenin/Tcf signaling by naringenin in AGS gastric cancer cell. Biochem. Biophys. Res. Commun..

[72] Park S., Chun S. (2011). Streptonigrin inhibits β-Catenin/Tcf signaling and shows cytotoxicity in β-catenin-activated cells. Biochem. Biophys. Acta.

[73] Voronkov A., Krauss S. (2013). Wnt/β-catenin signaling and small molecule inhibitors. Curr. Pharm. Des..

[74] Lepourcelet M., Chen Y.N., France D.S., Lubecka B., Matulewicz L., Maniakowski Z., Polaniak R., Birkner E., Rzeszowska-Wolny J. (2004). Small-molecule antagonists of the oncogenic Tcf/β-catenin protein complex. Cancer Lett..

[75] Barker N., Clevers H. (2006). Mining the Wnt pathway for cancer therapeutics. Nat. Rev. Drug. Discov..

[76] Trosset J.Y., Dalvit C., Knapp S., Fasolini M., Veronesi M., Mantegani S., Gianellini L.M., Catana C., Sundström M., Stouten P.F. (2006). Inhibition of protein-protein interactions: The discovery of druglike β-catenin inhibitors by combining virtual and biophysical screening. Proteins.

[77] Tian W., Han X., Yan M., Xu Y., Duggineni S., Lin N., Luo G., Li Y.M., Han X., Huang Z. (2012). Structure-based discovery of a novel inhibitor targeting the β-catenin/Tcf4 interaction. Biochemistry.

[78] Lu D., Liu J.X., Endo T., Zhou H., Yao S., Willert K., Schmidt-Wolf I.G., Kipps T.J., Carson D.A. (2009). Ethacrynic acid exhibits selective toxicity to chronic lymphocytic leukemia cells by inhibition of the Wnt/beta-catenin pathway. PLoS ONE.

[79] Jin G., Lu D., Yao S., Wu C.C., Liu J.X., Carson D.A., Cottam H.B. (2009). Amide derivatives of ethacrynic acid: Synthesis and evaluation as antagonists of Wnt/β-catenin signaling and CLL cell survival. Bioorganic Med. Chem. Lett..

[80] Moreira I.S., Fernandes P.A., Ramos M.J. (2007). Hot spots—A review of the protein-protein interface determinant amino-acid residues. Proteins.

[81] Young L., Jernigan R.L., Covell D.G. (1994). A role for surface hydrophobicity in protein-protein recognition. Protein Sci..

[82] Horton N., Lewis M. (1992). Calculation of the free energy of association for protein complexes. Protein Sci..

[83] Janin J., Bahadur R.P., Chakrabarti P. (2008). Protein-protein interaction and quaternary structure. Q. Rev. Biophys..

[84] D’Archivio M., Filesi C., Varì R., Scazzocchio B., Masella R. (2010). Bioavailability of the Polyphenols: Status and Controversies. Int. J. Mol. Sci..

